# MDMA-Assisted Psychotherapy for Cluster B Personality Disorders: A Narrative Review of This Translational Framework for Relational Healing

**DOI:** 10.1192/j.eurpsy.2026.11914

**Published:** 2026-07-31

**Authors:** M. Rocha, V. Sharma, S. Prasad, S. Gunturu

**Affiliations:** 1 American University of Caribbean School of Medicine, Cupecoy, Sint Maarten (Dutch Part); 2 Dayanand Medical College and Hospital, Ludhiana, India; 3 Psychiatry, BronxCare Health System, Bronx, United States

## Abstract

**Introduction:**

Psychedelic-assisted psychotherapy has re-emerged as a frontier of psychiatry, offering transformative potential for conditions resistant to conventional treatment. Personality disorders—particularly Borderline Personality Disorder (BPD)—are marked by affective instability, interpersonal dysfunction, and maladaptive relational patterns that often limit therapeutic progress. MDMA’s capacity to enhance empathy, reduce fear, and foster interpersonal trust may open a novel translational pathway for repairing disrupted attachment and self-structure in Cluster B disorders, integrating neurobiological, psychotherapeutic, and relational mechanisms within a unified model of healing.

**Objectives:**

To develop a conceptual and translational framework for the potential use of MDMA-assisted psychotherapy in Cluster B personality disorders, integrating neurobiological, psychotherapeutic, and ethical dimensions.

**Methods:**

A comprehensive search was conducted across PubMed, Scopus, and PsycNet using the terms *“MDMA,” “MDMA-assisted therapy,”* and *“personality disorders.”* No temporal or geographical limits were applied. After removing duplicates, articles were screened by title and abstract. Eligible studies included peer-reviewed original and review papers in English. Non-English publications, editorials, and opinion pieces were excluded. Additional sources were identified by reviewing reference lists of primary studies and conducting a targeted open-source Google search to capture conceptually relevant material.

**Results:**

MDMA acts as an entactogen, enhancing empathy, interpersonal connection, and emotional openness through serotonergic, dopaminergic, and noradrenergic modulation. These mechanisms may help correct the emotional dysregulation, diminished empathy, and relational instability central to Cluster B pathology. Two cohort studies (n = 21; n = 55) reported improved functioning and reduced anxiety, depression, and suicidality following psychedelic interventions. Conceptual models of MDMA-assisted therapy highlight increased prosocial affect and relational attunement, while early BPD trial designs suggest benefits in emotional regulation, reduced avoidance, and stronger therapeutic alliance, though empirical evidence in Cluster B disorders remains limited.

**Conclusions:**

MDMA-assisted psychotherapy may represent a promising adjunctive approach for Cluster B personality disorders by targeting deficits in attachment, trust, and emotion regulation. The proposed framework underscores the need for ethically grounded pilot trials, specialized therapist training, and robust safety monitoring. By bridging neurobiology and psychotherapy, MDMA-AT could advance integrative, relationally informed models for treatment-resistant personality pathology and encourage interdisciplinary collaboration within modern psychiatry.

**Disclosure of Interest:**

None Declared

